# Current concepts of perioperative monitoring in high-risk surgical patients: a review

**DOI:** 10.1186/s13037-019-0213-5

**Published:** 2019-10-23

**Authors:** Paolo Aseni, Stefano Orsenigo, Enrico Storti, Marco Pulici, Sergio Arlati

**Affiliations:** 1 Department of Emergency Medicine, ASST Grande Ospedale Metropolitano Niguarda, Piazza Ospedale Maggiore 3, 20162 Milan, Italy; 2 Department of Anesthesia and Intensive Care, ASST Grande Ospedale Metropolitano Niguarda, Milan, Italy; 3 Dipartimento Emergenza Urgenza, UOC Anestesia e Rianimazione, ASST, Lodi, Italy

**Keywords:** Hemodynamic monitoring, High-risk surgery, Oxygen delivery, Capnography, Ultrasound monitoring, Goal-directed therapy, Patient safety

## Abstract

A substantial number of patients are at high-risk of intra- or post-operative complications or both. Most perioperative deaths are represented by patients who present insufficient physiological reserve to meet the demands of major surgery. Recognition and management of critical high-risk surgical patients require dedicated and effective teams, capable of preventing, recognize, start treatment with adequate support in time to refer patients to the satisfactory ICU level provision. The main task for health-care planners and managers is to identify and reduce this severe risk and to encourage patient’s safety practices. Inadequate tissue perfusion and decreased cellular oxygenation due to hypovolemia, heart dysfunction, reduced cardiovascular reserve, and concomitant diseases are the most common causes of perioperative complications. Hemodynamic, respiratory and careful sequential monitoring have become essential aspects of the clinical practice both for surgeons and intensivists. New monitoring techniques have changed significantly over the past few years and are now able to rapidly identify shock states earlier, define the etiology, and monitor the response to different therapies. Many of these techniques are now minimally invasive or non-invasive. Advanced hemodynamic and respiratory monitoring combines invasive, non-invasive monitoring skills. Non-invasive ultrasound has emerged during the last years as an essential operative and perioperative evaluation tool, and its use is now rapidly growing. Perioperative management guided by appropriate sequential clinical evaluation combined with respiratory and hemodynamic monitoring is an established tool to help clinicians to identify those patients at higher risk in the attempt to reduce the complications rate and potentially improve patient outcomes. This review aims to provide an update of currently available standard concepts and evolving technologies of the various respiratory and hemodynamic monitoring systems for the high-risk surgical patients, highlighting their potential usefulness when integrated with careful clinical evaluation.

## Background

Intra-operative and post-operative complications in high-risk surgical patients remain significant causes of prolonged recovery, survival, and continue to represent a substantial proportion of ICU admissions in most developed countries.

All mitigation activities in the attempt to reduce these risks are essential not only for the individual patient safety but also for health-care managers.

It was estimated in 2008 that about 230 million surgical procedures were performed around the world [1], and a significant number of these patients were at risk of intra- or post-operative complications. Although less than 15% of inpatient procedures were performed in high-risk patients, such patients accounted for 80% of deaths [2–6].

Identification of these patients before surgical interventions remains a challenging task both in the emergency and elective surgical setting. The risk for death and severe complications in some high-risk patients after major surgery is mainly related to the patient’s pre-operative physiological condition and, in general, to their cardiovascular and respiratory reserves, as well as the type and the extent of surgery. Several technological advances with less invasive or non-invasive monitoring systems are rapidly growing. Concerns about training and safety use of these new devices are increasing, and appropriate teaching programs for surgical residents and fellows are highly desirable to encourage patient safety fundamentals in the trainee.

This narrative review aims to provide an update of current concepts about the appropriate use of the recent perioperative monitoring systems when combined with a clinically-based approach in the high-risk surgical patients.

### Search criteria

A literature review was carried through PubMed, Medline and Embase to identify any study on adults published from January 1988 to May 2019. The terms searched were “(HEMODYNAMIC MONITORING AND HIGH-RISK SURGICAL PATIENT) AND (CLINICAL MONITORING)” using “AND” as a Boolean Operator. The literature search identified a body of research with 175 relevant articles published in medical journals. We selected 51 articles from medical Journals including only articles in the English language, with a full-text availability, review articles, controlled clinical trials, clinical reports with more than 10 patients; some articles were excluded when they were redundant or poorly pertinent.

## The definition of “high-risk”

The term “high-risk surgical patient” is controversial and poorly defined and usually refers to patients, who are considered clinically to be at high-risk of peri and post-operative death; these patients can be selected to obtain a pre-operative “hemodynamic and clinical optimization”. Defining high-risk can be subjective and a variety of conditions can be considered such as surgical factors, complexity, the degree of urgency or emergency of the procedure, the skills and experience of the surgical and anesthesiological teams, the patient clinical status and comorbidities, the older age, as well as the availability of an appropriate and careful post-operative ICU management.

It has been suggested that patients with individual mortality risk of > 5%, or undergoing a procedure carrying a mortality risk greater than 5%, be defined as high-risk surgical patients; those patients for whom the probability for perioperative mortality is evaluated greater than 20% should be considered and defined ‘extremely high-risk’ surgical patients [7].

Some pragmatic assessments of pre-operative comorbidity have been employed by several investigators in the attempt to identify patients at higher risk of morbidity and mortality following surgery. In Table 1 are summarized some clinical conditions originally described by Shoemaker and then adapted by Boyd and Coll [8, 9]. which identify a cohort of patients at much higher risk than those in the general population of patients undergoing surgery.

**Table 1:** Clinical criteria for high-risk surgical patients (Reproduced with permission from Boyd O, Jackson N. Clinical review: How is risk defined in high-risk surgical patient management? *Critical Care* 2005, 9:390–396, Copyright Springer Nature)

Previous severe cardiorespiratory illness — acute myocardial infarction, chronic obstructive pulmonary disease, or stroke	
Late-stage vascular disease involving aorta	
Age > 70 years with limited physiological reserve in one or more vital organs	
Extensive surgery for carcinoma (e.g. oesophagectomy, gastrectomy cystectomy)	
Acute abdominal catastrophe with haemodynamic instability (e.g. peritonitis, perforated viscus, pancreatitis)	
Acute massive blood loss > 8 units	
Septicaemia	
Positive blood culture or septic focus	
Respiratory failure: PaO2 < 8.0 kPa on FIO2 > 0.4 or mechanical ventilation > 48 h	
Acute renal failure: urea > 20 mmol/l or creatinine > 260 mmol/l	

However, these criteria were open to some subjective interpretation and did not appear to be robust enough when they were applied in clinical trials; for this reason, they did not receive a wide application in the clinical practice. Although other tests were developed in the attempt to stratify the risk in surgical patients pre-operatively, the simplest and most widely used remains the American Society of Anaesthesiologists classification of the physical status (ASA PS) grading on a scale of I to VI. This simple method for assessing comorbidities is strongly associated with postoperative mortality, and it remains an excellent independent predictor of perioperative morbidity, and mortality [9, 10].

it was supposed that by targeting specific hemodynamic and oxygen transport during the perioperative period, the outcomes of these high-risk surgical patients could be improved. Goal-Directed Therapy (GDT) with the use of fluid loading and inotropes, to optimize the preload, contractility, and afterload of the heart while maintaining adequate coronary perfusion has been the object of several studies. Despite some benefits maintaining satisfactory oxygen transport and tissue perfusion were observed, there is no robust evidence to support that GDT may have substantially decreased the overall morbidity and mortality [11–17]. However, selecting the most appropriate hemodynamic and respiratory monitoring devices may provide a broader clinical picture to avoid both under, or over resuscitation, which are equally harmful and may be an essential step to reduce further complications [18, 19].

## Indications for essential clinical, respiratory and hemodynamic monitoring in the high-risk surgical patients

A careful clinical examination represents the most crucial initial step in the hemodynamic assessment of high-risk surgical patients. Basic hemodynamic monitoring usually includes a focused physical examination and vital signs such as temperature, respiratory rate, heart rate, mean arterial pressure, and arterial hemoglobin oxygen saturation, and urinary output. However, vital signs may lack the specificity and sensitivity to guide valuable hemodynamic management. It is often necessary to combine and integrate different parameters from different hemodynamic monitoring systems to significantly improve the understanding of hemodynamic status [6, 20]. For instance, the combination of arterial pressure and the partial pressure of end-tidal carbon dioxide (PetCO2) can help differentiate between vasodilation and low cardiac output (CO) as a cause of hypotension which may prevent futile fluid administration in a patient with a transient decrease of PetCO2 following a reduction of CO due to vasodilation. Similarly, a reduction in the PetCO2 for the same minute ventilation in the absence of hypothermia indicate a decreased pulmonary blood flow (and thus CO) and may suggest a more advanced hemodynamic monitoring [6, 21].

In hemodynamically stable patients, continuous electrocardiographic (ECG) monitoring, regular non-invasive blood pressure measurement, and peripheral pulse oximetry (peripheral oxygen saturation or SpO2) can be adequate.

In unstable patients or those who are at higher risk of hemodynamic instability, an arterial line for continuous invasive blood pressure measurement and analysis of arterial blood gasses at regular interval of time are recommended. Patients receiving vasopressors or inotropic agents requires a central venous line for drug administration. In some particular patients who fail to improve to initial resuscitation advanced or extended hemodynamic monitoring will be necessary to guide medical management [22, 23].

## Monitoring during anesthesia

Both surgery and general anesthesia induce changes in the patient’s physiology. The overall effects of drugs administered at anesthesia induction is to blunt consciousness and provide analgesia, but they also have other effects, such as reduction in muscle tone (even paralysis if a curare is administered), reduction in pulmonary functional residual capacity (FRC), decrease in CO, blunt of thermic homeostasis [21]. Moreover, respiratory drive suppression and careful and adequate patient monitoring are considered essential to titrate administration of anesthetic medication, to detect physiologic perturbations and allow intervention to prevent harmful conditions. The term “standard ASA monitors” is often used to refer to the basic physiologic monitors recommended by the American Society of Anesthesiologists and by the World Health Organization-World Federation of Societies of Anesthesiologists [20].

Both anesthesia and surgical incision cause an increase in stress hormone excretion (e.g., cortisol and catecholamines) leading to significant modifications in blood flow, increase in gluconeogenesis, protein catabolism, and peripheral leucocytosis [24]. During and immediately after surgery, depending on the type of surgical procedure and patient conditions, there is an increased risk of deep vein thrombosis and acute pulmonary embolism (PE).

In 1949, the New York Hospital Operating Room Committee stated categorically that “an adequate recovery room service is an essential requirement to any hospital undertaking modern surgical therapy”. After surgery, the high-risk surgical patient should be admitted to the post-anesthesia care unit (PACU) or recovery room [25].

## Methods of hemodynamic and respiratory monitoring

Several methods have been developed during the last decades. Some technologies for CO monitoring can be classified as calibrated or non-calibrated techniques or according to their level of invasiveness as invasive, less invasive, or non-invasive. There is now a general trend to use more less-invasive and non-invasive techniques to reduce the risks that accompany invasive procedures.

### Airway patency

Perioperative and post-operative airways collapse or occlusion may lead to hypoventilation, as decreased muscle tone due to anesthesia may cause tongue displacement or soft tissue collapse; laryngeal edema, laryngospasm or vocal cord paralysis may also develop. Reduced airway reflexes may cause gastric content aspiration and consequent pneumonitis [26].

Patients who underwent neck surgery, or in whom a laryngeal mask airway is placed, need closer attention.

Patient-related risk factors include male sex, age greater than 60 years, diabetes, obstructive sleep apnoea syndrome, and obesity.

### Respiratory function

Proper monitoring of respiratory function may help the early detection of hypoxemia and hypoventilation. Overzealous fluid resuscitation and reduced CO may cause pulmonary edema. Hypercoagulation and venous stasis are risk factors for developing a PE. During general anesthesia, a decreasing trend of the value of PetCO_2_ observed by capnography, and an increase in PaCO_2_ (separation phenomenon) can arise the suspicion of PE.

Pneumothorax can develop due to surgical penetration in the pleural space or procedures that can tear pleural wall such as a CVC placement.

Peripheral saturation, airways patency, respiratory rate should be measured in every patient. Patient at increased risk of developing respiratory complications should also have capnography, and hemodynamic status checked. Other respiratory monitoring techniques in high-risk surgical patients include arterial blood gas analysis, 12 derivations electrocardiogram, and imaging studies (e.g., ultrasound) [18, 19, 23].

### Cardiovascular monitoring

Cardiocirculatory dysfunction with subsequent hemodynamic instability is a frequent and crucial symptom in the high-risk surgical patients with reduced cardiovascular reserve. Hemodynamic instability diminishes oxygen supply to the end organs and is associated with an increased mortality rate.

Monitoring cardiovascular function is essential to avoid or to detect complications such as hypoperfusion, perioperative myocardial infarction, dysrhythmias, hypertension, hypovolemia, hyperthermia, hypoxemia, hypercarbia, bradycardia as a consequence of residual anesthetics effect, vagal stimulation, or increased intracranial pressure.

Basic hemodynamic monitoring comprises continuous ECG, pulse oximetry and noninvasive blood pressure monitoring [27].

Implemented hemodynamic monitoring requires 12 leads Electrocardiogram, vena cava assessment with US, Partial CO_2_ rebreathing, passive leg raise, and lactates.

Advanced or extended hemodynamic monitoring encompasses basic monitoring plus CVP and its derived measurements (ScvO2, Central venous to arterial carbon dioxide gap), transpulmonary thermodilution measurement of CO with all derived parameters of pressure (volumetric and dynamic preload, afterload and microcirculation parameters), pulse contour derived CO and functional hemodynamic monitoring, gastric tonometry, thoracic bioimpedance, thoracic bioreactance, and transoesophageal echocardiography.

#### Basic cardiovascular and hemodinamic monitoring

Includes continuous ECG, pulse oximetry and Blood pressure (BP)**.** BP is an easy and usually non-invasive parameter to be checked. Mean arterial pressure (MAP) > 70 mmHg is generally considered to be an index of good tissue perfusion. MAP is often used as an index of CO. However, blood pressure is physiologically auto-regulated, and its measure cannot predict whether the patient is hemodynamically stable, and he or she is compensating an impending unstable hemodynamic situation since others variable influencing CO could change without any appreciable alteration in BP. Due to this, low CO states, including hypovolemia, can present with BP within the normal range, as a result of increased peripheral vascular resistance. Hypotension is a late sign of low tissue perfusion, and it reflects a failure in compensatory mechanisms which usually counterbalance the hemodynamic collapse [12, 17, 19, 28]. Shock index (SI) is defined as the heart rate (HR) divided by systolic blood pressure (SBP). It has been studied in patients either at risk of or experiencing shock from a variety of causes such as trauma, hemorrhage, myocardial infarction, pulmonary embolism, sepsis. However, SI has some disadvantages since it appears normal in the compensatory phase of shock and can be confounded by factors such as medications (e.g., antihypertensives, beta-agonists). SI > 1.0 has been found to predict an increased risk of mortality and admission to intensive care units [29].

#### Intermediate hemodynamic monitoring

-12 leads Electrocardiogram (ECG). Monitoring electrocardiogram in the perioperative period in high-risk surgical patients is among the foremost recommended standards. Some other information other than the cardiac status is possible from ECG signals such as respiratory rate monitoring and ventilator triggering. Application of advanced technology in ECG monitoring gives maximum information and should be utilized to its fullest extent in high-risk surgical patients [30].

-Vena Cava Assessment. Vena cava can be assessed with US and distensibility index of inferior vena cava in mechanically ventilated patients can be used to predict fluid responsiveness accurately [11–13]. This technique is poorly reliable in spontaneously breathing or with high intra-abdominal pressure patients, and may be difficult to obtain in the obese ones.

-CVP*.* Central venous pressure should not be considered as an index of fluid status: many studies have shown no correlation, mainly if used as a static index [31].

-Central Venous oxygenation (ScvO_2_); a *ScvO*_*2*_ < 70% shows early patient hemodynamic imbalance, before other hemodynamic or lab values changes. When correctly measured ScvO_2_ is considered a valid endpoint for shock resuscitation and a ScvO _2_ between 70 and 89% would suggest an adequate balance between oxygen demand and oxygen supply (VO _2_ /DO _2_) [7, 18, 19, 29].

**-**Central venous to arterial carbon dioxide gap (dCO_2_); dCO_2_ has been proven to be a good and early index of tissue perfusion imbalance in the critically ill patients [25]. Furthermore, if the impairment is confirmed with low ScvO_2_ an improvement in its specificity, positive and negative predictive values have been reported [32].

**-**Partial CO_2_ rebreathing; This technique is accomplished using a specially designed breathing system that allows brief (50-s) increase of dead space. In this manner, CO_2_ is rebreathed, allowing the use of the Fick equation on its minimum and maximum values. This technique allows safe and non-invasive measurement of CO, but it is applicable only in intubated patients [18–20, 23].

*-*Lactates**;** Lactate clearance can be used to monitor tissue perfusion [12, 17]. Normalization of lactate level is considered a valid endpoint for shock resuscitation, mainly if a trend is used rather than its static value [33].

-Passive leg raise (PLR)**;** Fluid responders are defined as those patients that increase stroke volume more than 10% after a fluid bolus (e.g., fluid challenge); PLR is a maneuver that mimics fluid challenge without actually giving any fluids. PLR should be started with the patient in semi-recumbent position: it is performed lowering the trunk while raising the legs. Positive prediction of fluid-responsiveness [34, 35] should be assessed with CO or stroke volume measurement (threshold: a raise between 8 and 15%), or, if unavailable, radial pulse pressure (threshold: an increase between 9 and 12%).

#### Advanced hemodynamic monitoring

-Pulse contour derived CO and functional hemodynamic monitoring**.** Recently, several monitors enable to follow track changes in arterial pressure non-invasively from finger probes. These include the continuous non-invasive arterial pressure probe. These monitors have the potential to track the stroke volume (SV) and CO in situations requiring early hemodynamic intervention when more invasive monitoring modalities are not readily available [12, 36]. Functional hemodynamic monitoring is the measurement of the hemodynamic response to a predetermined intervention; it may predict volume responsiveness, arterial vasomotor tone reactivity (elasticity), and microvascular tissue hypoxia due to cardiovascular insufficiency, even in the setting of compensated shock.

By using these systems, a patient is defined as “not fluid responder” with a Stroke Volume Variation (SVV) less than 10% and a pulse pressure variation (PPV) less than 9%, and “responders” with SVV major than 14% and PPV greater than 13%. The areas between these values are considered grey areas, in which these systems are not able to provide reliable information. Various commercial devices are now available to calculate and continuously display PPV, stroke volume variation (SVV), and cardiac index [36, 37].

-Gastric tonometry (GT); Systemic parameters of tissue oxygenation are not reliable enough to represent “true” tissue oxygenation status, and gastric tonometry represents a new approach to the problem. GT is the measurement of gastric intramucosal pH (pHi) with a gastric tonometer. This device consists of a modified nasogastric tube into the stomach attached to a silicone balloon that is filled with saline, equipped with an indicator. By GT, it is possible to measure gastric intramucosal pCO_2_ and then, calculate, by using Handerson-Hasselbach equation, intramucosal pH (pHi). Several clinical investigations show that pHi is a sensitive and specific prognostic marker. Gastric pH behaviour can predict multiorgan dysfunction and mortality in different groups of critically ill patients. Its use to guide resuscitation maneuvers could contribute to mortality reduction [38].

-Transpulmonary thermodilution of CO*;* This technique is considered a gold-standard in measuring CO. Although quite invasive (a central line and an arterial line are required), it can give accurate and valuable information.

The measure of the CO is based on the Fick principle [CO = VO_2_/ (CaO_2_-CvO_2_)] where VO_2_ is the consumption of oxygen and CaO_2_, and CvO_2_ are the arterial and mixed venous oxygen contents, respectively. Arterial and mixed venous oxygen can be measured by using blood samples from a peripheral arterial line (oxygenated blood) and a central venous catheter (deoxygenated blood), respectively. This method is invasive and time-consuming, and although considered the gold standard for CO, it is seldom utilized in ICU. Measuring CO and its components (preload, afterload, and contractility) can give information about the ongoing need for fluid resuscitation, vasopressors, or inotropic agents. Furthermore, it can be used to guide de-resuscitation, the phase during which there are often some risks of fluid overload (which is itself an important adverse prognostic predictor [14, 20, 22, 23].

-Thoracic bioimpedance; While thought to be inaccurate, more recent studies addressed this technique to be reliable. By using low-voltage electrodes on the chest wall, electrical impedance is measured. Since fluids offer less resistance to electric flow, blood volume changes can be measured [39, 40].

-Thoracic bioreactance; Bioreactance is a technique based on bioimpedance: by using the same electrodes, it measures the phase shift in alternating current voltage across the thorax. The phase shift is determined for the most part by pulsatile flow (e.g., blood flow) rather than static fluids (e.g., intravascular and extravascular fluids), therefore improving bioimpedance by removing signal noises. The measurement of thoracic bioreactance may improve fluid balance, time needed in mechanical ventilation, ICU length of stay, need of haemodialysis, and time on vasopressors [41].

-Transoesophageal echocardiography (TEE); TEE is an essential cardiovascular diagnostic tool***,*** which is strongly operator dependent. The US real-time images of the cardiac structures and blood flow are provided by the transducer which is placed in the esophagus next to the heart. It may help define pathophysiological abnormalities in patients like wall motion abnormalities, pericardial effusions, pulmonary hypertension, and valvulopathy, in conjunction with other invasive or less-invasive monitoring. A significant learning curve is required for TEE, which is also expensive [42].

## Clinical monitoring of perioperative and postoperative complications

The mainstays of postoperative care are regular assessment, and selective clinical monitoring of the major body systems, namely respiratory, cardiovascular, and renal systems. All vital signs should be recorded as well as all signs of bleedings, dehydration and hypoperfusion (diuresis, core temperature, fluid and blood spillage from drainages, mucosal appearance, jugular veins filling or capillary refill) and particular emphasis should be addressed to detect early signs of sepsis [6, 7, 18]. Specific vital sign abnormalities in elderly patients such as systolic blood pressure < 97 mmHg, heart rate > 101 beats per minute, hyperthermia or hypothermia < 36 °C, pulse oximetry < 92 SpO_2_ are consistently associated with adverse patient outcomes [9, 21, 43].

Proper monitoring of patient status can guide therapy for appropriate fluid therapy when shock is a risk. The European Society of Anaesthesiology encourage GDT, as it decreases postoperative complications rate and length of stay and early GDT can reduce mortality in septic shock in the severe sepsis settings [44–46].

Postoperative complications greatly depend on the type of surgery*.* Gastrointestinal complications are more frequently seen in patients undergoing abdominal surgery. Patients at risk for gastrointestinal complications include patients with coagulopathies, perioperative impairment of tissue perfusion, spinal cord injuries, severe burns, hepatic or renal failure, polytrauma, organ transplantation, need for prolonged mechanical ventilation, hypercoagulable states, hypovolemia. Following abdominal surgery, postoperative ileus can be observed. Depending on the gut segment involved, it may last up to 72 h, and time to first solid food intake must be planned accordingly [47]. Some patients can be at risk of endocrine complications such as thyrotoxicosis in patients with previously unrecognized hyperthyroidism. Hypertensive crisis can be observed in patients with unrecognized pheochromocytoma. Acute adrenal insufficiency or adrenal crisis can develop in patients with adrenal insufficiency or in those who are in steroids therapy but have not increased the dose of glucocorticoids, as well as in patients without a prior diagnosis that have encountered acute physical stress. Adrenocortical insufficiency should be suspected in the event of hypotension in patients with chronic glucorticoid therapy who does not respond to fluid replacement and vasopressor agents [48].

### Perioperative and postoperative UltraSonographic evaluation

Bedside or Point of Care UltraSonography (PoCUS) is rapidly becoming the new standard monitoring device for real-time diagnostic assessment in addition to clinical and standard physical examination in the emergency department, intensive care unit, and now in the perioperative and postoperative period [49].

PoCUS is a diagnostic modality that provides clinically significant data usually not obtainable by inspection, palpation, auscultation, or other components of the physical examination, and should be considered complementary to the physical examination. Bedside imaging may provide to the anesthesiologists the additional information about the volume status, basic cardiac function, lung status, and respiratory function, and It is fundamental for early detection of intrabdominal and intrathoracic bleeding or fluid collection. PoCUS, when used for airway and lungs assessment, allows careful clinical evaluation of airway patency and effective breathing also in emergency conditions. In the daily practice, it can help in the evaluation of patients in spontaneous and artificial ventilation guiding the intubation maneuvers and granting dynamic lung assessment.

**Fig. 1:**
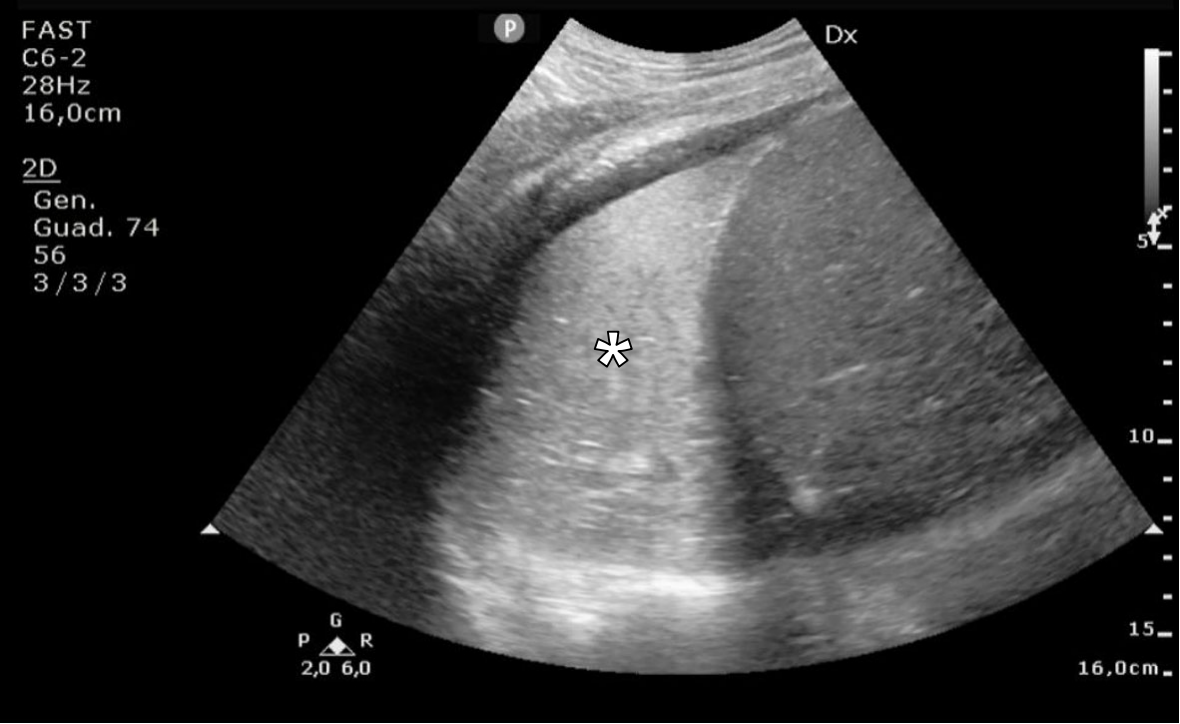
Early post-operative atelectasis: longitudinal US scan of the thorax at the level of the mid-axillary line, obtained with a convex probe, showing (white asterisk) an extensive area of lung consolidation

**Fig. 2:**
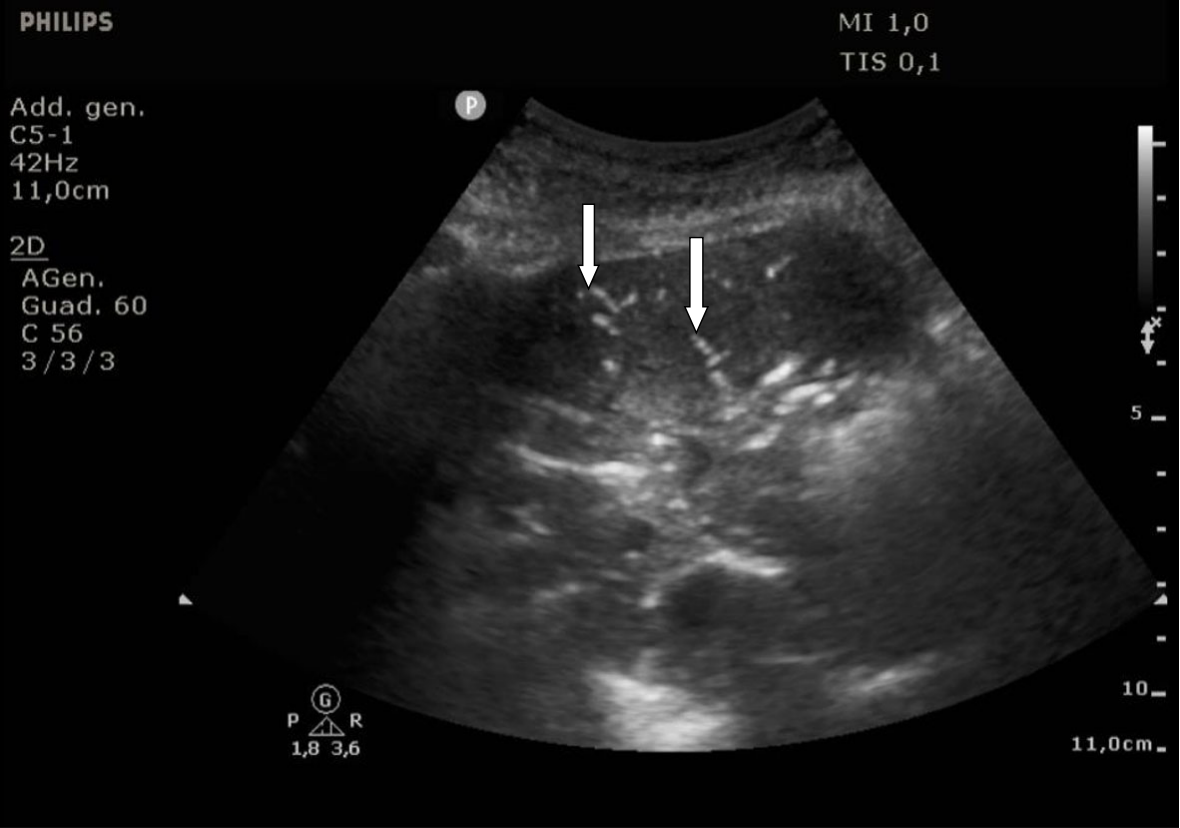
Sonographic air broncograms. The longitudinal US scan of the thorax, obtained at the level of the mid-axillary line with a convex probe, shows a large post-operative pneumonia. Two air-bronchograms are visible as two linear hyperechoic findings (white arrows)

**Fig. 3:**
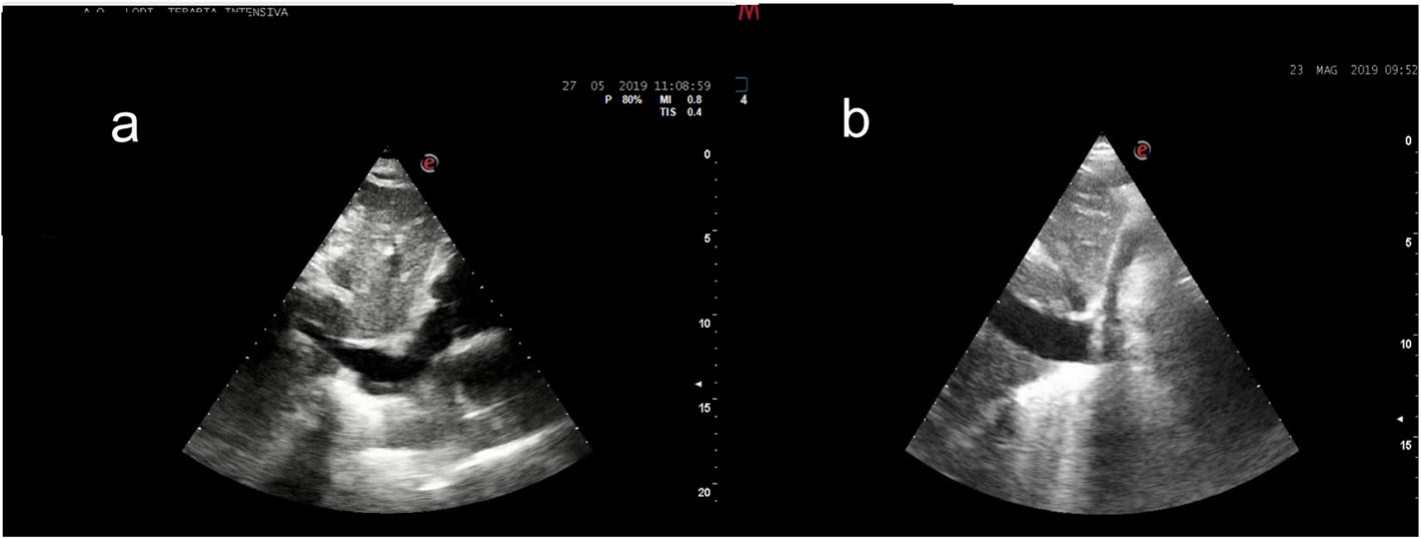
Subxiphoid longitudinal window allows to easily and quickly visualize the IVC. On the left side (**a**) the IVC in an ipovolemic patient is totally collapsed, on the right side (**b**) is shown the IVC in a patient with a hypervolemic conditions (distended, not collapsing)

Using a combined ultrasonographic evaluation of multiple districts (inferior vena cava, heart chambers, and lung parenchyma), it is possible to determine the hemodynamic status of the patient and to assess its changes in relation to the treatment performed. PoCUS, for its intrinsic characteristic of being fast, reliable and easily replicable, it is particularly useful for the periodic assessment of relevant clinical variables; it can be used as a valid instrument to constantly monitor the patient’s status, anticipating potential detrimental evolutions thereby configuring itself as a precious tool guiding in the correct management of critical patients (Figs. 1, 2 and 3). Using bedside ultrasound imaging in an assessment of gastric contents has recently been reported as a useful and easy method which supports an objective, quick evaluation of the risk of aspiration and could be particularly interesting in the emergency setting [50].

## Conclusions

The classical measures of hemodynamics, often referred to as routine vital signs, are central to the assessment of cardiorespiratory sufficiency and most of diagnostic medicine is based on bedside diagnostic tools (pulse oximetry, systolic and diastolic blood pressure, ECG) and some simple “human instrument” measures such as a careful inspection of the patient. The health care professionals represent the simplest form of early monitoring with the clinical inspection of the patient, if he is conscious, agitated or in distress, observing the patient’s breathing if regular or not, the presence or absence of central and peripheral cyanosis, if its skin is cool and moist, evaluating the central and peripheral pulses, if the capillary refill is rapid or not [51]. However, these simple and inexpensive measures do not always have a predictive value in identifying patients as being stable or unstable when compensatory changes in physiologic state are rapidly occurring above all in the high-risk surgical patients who are characterized by a reduced cardiorespiratory reserve. Although there are no ideal hemodynamic and respiratory monitoring methods that can provide accurate, reproducible, and reliable information on all parameters of the respiratory and cardiovascular system, a multitude of invasive, less invasive, and non- invasive techniques are now available. For everyday use in the clinical practice, the diversity of minimally invasive hemodynamic monitoring requires appropriate knowledge of several different techniques available, their settings, and their potential clinical validity. During de-resuscitation, the monitoring technique should be re-evaluated, and non-invasive techniques should be used whenever possible instead of invasive procedures. The improved ability to identify the high-risk surgical patients may help to ensure that limited, and sometimes expensive perioperative monitoring and intervention resources can be allocated to a subgroup of surgical patients who are most likely to receive a real benefit.

## Data Availability

Not applicable.

## Abbreviations

ASA PS: American Society of Anaesthesiologists classification of the physical status
CO: Cardiac output
CVP: Central venous pressure
GDT: Goal-Directed Therapy
GT: Gastric tonometry
GT: Gastric tonometry
PACU: Post-anesthesia care unit
PetCO2: Partial pressure of end-tidal carbon dioxide
PoCUS: Point of Care UltraSonography
PPV: Pulse pressure variation
SV: Stroke volume
SVV: Stroke Volume Variation
TEE: Transoesophageal echocardiography
